# Exposing the benefits of a pedagogy of partnership in health professions education

**DOI:** 10.4102/hsag.v28i0.2329

**Published:** 2023-09-22

**Authors:** Penelope Engel-Hills, Hilde Ibsen, Llizane McDonald

**Affiliations:** 1 Faculty of Health and Wellness Sciences, Cape Peninsula University of Technology, Cape Town, South Africa; 2 Department of Political, Historical, Religious and Cultural Studies, Faculty of Arts and Social Sciences, Karlstad University, Karlstad, Sweden; 3 Department of Emergency Medical Care, Faculty of Health and Wellness Sciences, Cape Peninsula University of Technology, Cape Town, South Africa

**Keywords:** pedagogy in context, community-based service learning, responsive curriculum, reciprocal learning, social justice, social consciousness, first aid training, relevant curriculum

## Abstract

**Background:**

Literature indicates the need to prepare health professionals who are clinically competent and socially conscious. Engagement in community projects, as an extension of workplace learning, can build professional competence and social awareness.

**Aim:**

To interrogate one such engagement; an emergency first aid responder training course was orchestrated by undergraduate students studying Emergency Medical Care.

**Setting:**

The intervention was offered in response to a community need emerging from the research project being conducted in a community in the Western Cape, South Africa.

**Method:**

Qualitative data were gathered as narrative texts from participants in the intervention and student reports about their learning experience. The data were interrogated through the application of reflexive thematic analysis and the theoretical lens of asymmetrical reciprocity.

**Findings:**

The three themes that emerged were: from research to a student led intervention, deep authentic learning, and learning as a shared experience. Benefit accrued to the students and community through a partnership of asymmetrical participants. The community offered a learning experience while students offered desired skills acquisition to community members.

**Conclusion:**

Through this interaction, students learnt respect for local knowledges, and gained enhanced social awareness, in a transdisciplinary partnership, that aimed to create a learning environment where academics, students, and community members are partners in a project delivered with a core value of social justice.

**Contribution:**

A pedagogy of partnership describes an education model arising from community-based research that enabled a social programme intervention as a relevant learning project for health science students.

## Introduction

Agenda 2063: The Africa We Want, was launched by the African Union Commission in 2013. This agenda presents a call for social justice and development within the ethical paradigm of human rights (African Union Commission 2015). Furthermore, it is a plan for transformation, with the focus on honouring the rich heritage and culture of the continent, while anticipating growth of African knowledge and participation of all citizens in shared decision making in an inclusive society. Education is a main concern, and it has been acknowledged that the lack of human capacity could hamper the realisation of the Agenda 2063. Important current issues include the notion of decolonising the curriculum; first brought into focus in the student led ‘Rhodes Must Fall’ campaign in 2015 (Matebeni 2018). According to Essop (2016), decolonisation involves more than superficial curriculum changes and to effectively address the long-term effects of colonisation there needs to be revision of the university system and changes to the higher education (HE) culture. Another issue stressed by Essop (2016) is the need to broaden the debate on curriculum reform beyond the limitations of a decolonisation lens. This thinking influences current concerns in health professions education (HPE) where there is the quest to advance social justice in the curriculum to prepare students ‘to become change agents’ of the future (Jacobs et al. 2020:112).

This article contributes to the academic conversations about how the university can engage to transform the learning environment through changes to teaching and learning practices. The goal was to enable a relevant service learning (SL) activity by taking account of the needs of the community. The aim of this interrogation was to gain a deeper understanding of the experiences of the students and community members involved in a community-based service learning (CBSL) project in the local community of Ocean View, and to explore ways in which the pedagogies and practices in HE can transform HPE and student learning. In this article we will provide a background to the partnership, address why Ocean View was selected for this curriculum intervention and why the first responder course was viewed as relevant. The literature informing this study will then be presented, followed by the methodology and method of the Emergency First Aid Responder (EFAR) programme intervention, which is central to this article. Thereafter, the findings will be presented as three themes, followed by a discussion to draw attention to key points, and finally some conclusions that include suggestions and recommendations.

## Background

The partnership in the study presented here emerged from the joint South Africa-Sweden collaboration programme aimed at strengthening ties between the two countries in research and HE. The key partners in the project, *Internationalisation for knowledge partnership and social transformation* (IKPST), are one university in South Africa and another in Sweden, a non-profit organisation (NPO) in the community of Ocean View in the Western Cape, South Africa, and residents of this community. The objectives of this collaboration included a contribution to capacity building and social transformation within the particular community.

Notwithstanding having acknowledged the United Nations Sustainable Development Goals 2030 as a framework for sustainable development and being committed to becoming a safe, caring and inclusive place for all, the City of Cape Town is associated with high levels of unsafety (Kynoch 2016). Arguably there is a strong connection between the high levels of urban violence and Cape Town being the most segregated city in South Africa, with massive socio-economic inequality (Oxfam South Africa 2020). In many communities in South Africa, such as Ocean View, that were established through the apartheid past (Maralck & Kriel 1984), people frequently talk about empty promises and frame a narrative about politicians and organisations who do nothing for them (Buthelezi 2012). Betrayed promises arguably imply the persistence of unjust structures and of neglect, which according to Johan Galtung is a subtle form of violence, conceptualised as ‘structural violence’ (Galtung 1969:167–191). Pillay (2008) describes structural violence as being those routine and everyday occurrences of violence that are manifest in South Africa as normalised violence (Pillay 2008).

The two researchers were introduced to the community of Ocean View, approximately 35 km from the city of Cape Town, and an NPO in this community who were willing to support research and interventions. The project was focused on the challenges within the community of Ocean View, which included being disconnected from economic drivers, low employment opportunities, and structural violence. The study explored the lived experiences of residents, facing frequent episodes of violence and living with fear as a constant reminder of unsafety. From numerous conversations it became obvious that people wanted to be able to take action following traumatic incidents; but the participants expressed that they felt helpless in a medical emergency. Because of the South African principal investigator (PI) being at a university of technology (UoT) that has a Department of Emergency Medical Sciences (EMS) and where students, in their second year of study, on the Bachelor of Health Science: Emergency Medical Care (EMC), are required to conduct a community-based service learning (CBSL) project as part of the module. Primary Health Care options were opened to offer an appropriate intervention. While participation in one service learning (SL) activity is compulsory for the Primary Health Care module, the students have an element of choice as to which project they become involved in. In 2019, the project option for students to plan and offer the EFAR programme in the community of Ocean View was introduced. This programme was offered with the guidance and support of the coordinator of the EFAR system, developed as a cost-effective community-based prehospital emergency service in this province (Slingers, De Vos & Jared 2022).

The NPO agreed to provide facilities over weekends and two second year students willingly chose to lead this project. They formed a team of students from their second-year peers, who all had to be involved in one or more SL project, and they also included students from other year groups who volunteered their time to assist. In this process, the dual aims of the IKPST project – to build knowledge partnerships and enhance social transformation, and further to contribute to capacity building in our students – were woven together. We could not plan for this in advance, but the community-based research (CBR) raised a need that could be fulfilled by students through a SL project that provided them with an opportunity to gain experience in an authentic setting. Within this context, our research question was: how can the university transform the learning environment to increase access to relevant social experiences for HPE students?

## Ethical considerations

Ethical approval and site permission was granted through the university and community structures. A core understanding of ethical research in this environment was that ethical listening increases agency (Beard 2009), and this became the guiding framework for engagement. The principles of informed consent were followed and residents and students voluntarily engaged in the EFAR programme. They were advised that they could leave at any time without consequence, although it is observed that all the residents who started the programme were keen to complete. The ethical standard of confidentiality of individuals is adhered to in this article for students and residents. This project was considered to be of negligible risk above those of daily life for the participants, but at all times care was taken to monitor and mitigate against possible risks.

## Literature informing the study

This study makes a contribution to academic conversations about how the university can engage in ways that transform the learning environment (Leal Filho et al. 2018). It is informed by the writings of scholars that demand of the researchers to act with moral agency, within a paradigm of social justice and awareness (Shore & Freire 1987), and to respond to the crisis in HE in Africa in a context of transitioning to responsive curricula and the creation of transformative learning spaces (Jacobs et al. 2020). A responsive curriculum is relevant and more meaningful, but it does not take away from the essential core professional competencies while providing experiences that make graduates more critically conscious (Freire 2000). In this project the intention was to invoke social change by ‘changing our thinking and doing’ (Du Plessis 2016:vii) and to develop health professionals who are more able to appreciate the fabric of an unequal society and who have a clearer understanding of aspects such as the social determinants of health and well-being. As indicated by Shore and Freire (1987), transformation towards social justice takes place in the dialogical process of including students as active participants and co-creators of their learning experience (Morieson et al. 2018) as they uncover problems and solutions in a local community (Shore & Freire 1987).

Influenced by the student protests from 2015 to 2017, Mbembe (2019) addresses the call for conscious development. Luckett and Shay (2017) build a similar argument and suggest that to address the inequalities in South Africa, re-structuring is needed to create a transforming and inclusive system that asserts HE as socially just and that brings society more overtly into the curriculum.

The overarching pedagogy in place for the health science programme under consideration is work-integrated learning (WIL) that can include pre-entry workplace experience, apprenticeships, learnerships, experiential learning, and post-qualification internships (Reinhard et al. 2016). In the context of this article, WIL is the term that describes an approach to professional education that allows for the inclusion of theoretical forms of learning, problem-based learning (PBL), simulated learning, project-based learning (PJBL), workplace learning (WPL) and more (Winberg et al. 2011). Work-integrated learning, as it is applied in the EMC programme, is the explicit focus on integrating forms of learning to emphasise and build professional competence. It is an educational approach that achieves strong alignment between the academic components of the course, the simulated work environments and skills laboratories at the university, and the real world work placements and SL activities that students engage in during their time on this programme. This raises SL as another educational concept that applies to this study and which can encompass a wide range of activities by students in a variety of contexts. The understanding that is drawn on is of SL as a type of experiential learning that entails the provision of a service to meet an identified need in a specific community and that by its nature is based on reciprocity, such that the student and the community both benefit from the experience (Mouton & Wildschut 2005). The key elements of SL that apply to the EMC programme are that: the service provision is closely aligned to the curriculum content, the inclusion of the CBSL translates into relevant learning, and there is simultaneous empowerment of the community (Mouton & Wildschut 2005). Community-based service learning in the context of this study is the delivery of the EFAR programme in the community as an integral component of the WIL curriculum.

## Theoretical underpinnings

Being in a community, can for students, resemble site-based experience for the promotion of learning in context (Choy, Kemmis & Green^2016^). Site-based learning was helpful to think through this one example of a multipartner collaboration, located within the basket of workplaces and experiences that students in health sciences must have access to in order to prepare them as novice practitioners. South Africa needs healthcare practitioners who can appreciate the influence of the complexities and asymmetries of society on their professional practice. The process must however, go beyond being experiential such that student learning is internalised and influences their behaviour as professionals. To achieve this, students need to engage in reflective practice (Schön 1983), which was incorporated into the design of the intervention to enhance their learning and understanding of the EFAR experience.

Following thematic analysis, asymmetrical reciprocity (AR) (Young 1997) was applied as a theoretical lens to gain a deeper understanding of the factors that enabled or constrained the success of the intervention in this community setting. According to Young (1997), AR helps to gain an understanding of people by taking account of their differences during a reciprocal process of engagement that seeks to establish a relationship of equals working towards a mutually beneficial outcome. Asymmetry in this perspective is the involvement of persons with vastly differing histories, life experiences and social positions, and reciprocity captures the respectful characteristics of the ongoing research relationships where the parties strive to establish and maintain equality in the engagement (Silbert 2019; Trimble & Fisher 2006). Asymmetrical reciprocity facilitated the desire to demonstrate to the students a shift from the more typical position of expert to that of contributing to a reciprocal social interaction (Boser 2006).

## Methodology and method

This article arises from research in Ocean View between February 2017 and November 2019. Our approach was to engage in the co-creation of knowledge, as we focused on the establishment of a more equal relationship between the university and community to enable relevant research. The longitudinal study that evolved this article presents the research-initiated intervention of offering the EFAR programme. This CBSL project modelled the shift towards a more inclusive society for our students as the intervention sought to enable reciprocal relationships between the academics, researchers, students, and community members collaborating on the EFAR programme. The activity was isolated for interrogation because of the curriculum and pedagogical lessons that contribute to answering the research question: how can the university transform the learning environment and increase access to relevant social experiences for students? The intervention can be viewed as a transdisciplinary study that was designed to consciously reduce power relations and build trust by interweaving diverse stakeholders and partners (Mobjörk 2010). There were two researchers as the PIs from HE; one in health sciences at a UoT in South Africa and the other from social science at a university in Sweden. There were several academic staff members from the same South African UoT as the one PI who contributed with one academic who took on the responsibility of overseeing this SL project being a key collaborator.

The two students in EMS who coordinated this intervention and became the EFAR trainers were second year students because that is the year when SL projects are included in the curriculum. Both students were female and in their early 20s. The functions these two students took on during the planning and preparation stages included: liaison with the coordinators of the EFAR programme for the Western Cape, South Africa, communication with the NPO regarding access to the venue, connecting with the research assistant (RA) in Ocean View who set up the groups for each training day, coordinating the shopping for the refreshments and all preparations related to selecting the correct equipment, such as training manikins, and packing the transport vehicle as well as coordinating travel arrangements for all the students. The activities at completion of the programme included liaison with the external university who as the coordinator of the EFAR programme; moderated the assessments and issued certificates to successful candidates. Arrangements were made for a remedial course and reassessment in order that as many people in the community as possible could be successful, receive certificates, and be available when first aid was needed. The student leaders also coordinated arrangements and catering for a certificate ceremony held in a church hall in Ocean View.

The additional students in the team included five other second year students, two first year students, and a third year student to make up the eight students who contributed data to this project. These additional students helped with all the preparations before the training sessions and on the day were allocated to kitchen duty, childcare, administration functions for EFAR registration and assessment, and assisting with hands-on practice on the manikins. Five were female and there were three men among this cohort. All the activities of the students were supervised by academic staff members in the Department of EMS and the academic staff member presented at each training session. This academic drove the university emergency vehicle with all the equipment and supplies to the venue and was on hand to monitor the training provided.

The significant community partner was appointed as a RA on the larger project. She has lived in Ocean View because her family was relocated there during the forced removals in the 1970s. The RA is married, in her 40s and lives in Ocean View with her husband, son and his family. She is an involved and active resident, with in-depth local knowledge and a wide contact base that contributed to the success of the project. Sophie took on the role of identifying and communicating with the EFAR learners who were all residents of Ocean View.

Aligned to the ethos of the EFAR system, trainees are laypersons who are provided with skills and knowledge to allow them to take action when emergencies arise in their community and who support the prehospital emergency care teams as first responders (Slingers et al. 2022). There were four training sessions conducted over a full day. The participants in the four groups numbered 22, 19, 19 and 12 respectively, for a total cohort of 72 participants. The age range of the participants was between 22 and 63 years old with the majority in their late 30s and 40s. The majority were women with eight men participating overall. It is observed that the children who attended were not part of the training or the research. They were there to be looked after so that their parents, mostly the mothers, could attend the training. No records were kept of the children present.

In the design there was the simultaneous integration of capacity building for members of the community, as recipients, and students, as presenters and enablers of the EFAR training. It is reiterated that this programme could be offered only because of the established research partnership that created an opportunity for a relevant social intervention. A qualitative, interpretive approach was chosen for this study because a rich understanding of the lived experiences of the community participants and the students was sought (Terre Blanche, Durrheim & Kelly 2006). The research on community challenges and responses spoke to the aim of the researchers to contribute to social transformation through seeking practical solutions to social ills and structural injustice (Collins 1998:xiv) and further, the methodology reflects the call for co-learning through people’s priorities and a diversity of knowledge traditions (Mbembe 2019). The use of collaborative methodologies for the co-creation of knowledge (Facer & Enright 2016) encouraged community residents and university members to work in partnership, value one another, and engage on equal terms while acknowledging and respecting traditional and local knowledges.

The research methods took a less formal participatory observational style that enabled the researchers to be more connected to the participants throughout the data gathering process. This meant engaging in conversation with the 10 student and 72 community participants, as a form of interview, while sharing an activity. This approach deepened the researchers’ understanding of the social, cultural, and historical local context, and allowed for richer accounts of perceptions and subjective experiences (Miaux et al. 2010). The authors were therefore present and engaged in the lived experiences of the students and community participants during all the stages of the EFAR programme.

The data for this study were generated during the first semester of 2019, with the first EFAR course offered in April 2019 and another three courses following thereafter. In the dynamic collaborative approach, we had planned data collection points, such as the review of formal records related to the EFAR programme and the SL project and requested participant reflections. We also allowed for spontaneous contributions in the form of conversation and gathering of participant comments during the entire intervention.

The text data therefore came from a number of sources including: field notes and records of the PIs that were drafted from the review of planning documents related to the EFAR course and in real time during the four training sessions, verbal conversations with students and residents during the training offered over several weekends, written resident commentary in the form of a course evaluation, student generated reflections documenting their learning experience as facilitators and contributors, verbal input from participants at the conclusion of the intervention when certificates were presented to successful recipients, and finally from the content of the student reports on the project and their presentation on the delivery of the EFAR training in Ocean View at the class SL presentation day as part of the assessment of the SL projects.

All text data were analysed through the approach of reflexive thematic analysis to enhance critical reflection while uncovering and analysing the themes (Braun & Clarke 2022) that are reported on here. During the six-step analysis process of becoming familiar with the data, generating initial codes, developing and reviewing themes, naming these themes and producing a report (Braun & Clarke 2022), the critical reflection included scrutiny of the data and emergent themes through the theoretical lens of AR (Silbert 2019). In this phase, we searched for an indication that student participants were respectful and thought about issues from the perspective of the community participants. It was necessary to see if evidence existed that the students used this to decide on actions as just or right, were moved to being less focused on self, and could appreciate similarity and difference between themselves and the community members. This analysis was focused on seeking evidence of students recognising asymmetry in the relationship, observing ways in which the students attempted to mitigate the asymmetry, and whether they were able to recognise the reciprocity of learning in a space where they were simultaneously learning and facilitating learning for the EFAR participants. Students engaging in reflection, as a cornerstone of reciprocity, were encouraged within the structure of the intervention.

## Findings

The aim of this study was to explore the factors within two intersecting systems: university and community, which allowed for the transformation of the learning environment so as to increase students’ access to relevant social experiences. In doing this we sought to answer the question: how can the university transform the learning environment and increase access to relevant social experiences for students?

Lessons were gleaned from the data and reflections on the data representing the lived experiences of students and residents, shown as S and residents, shown as R. In each F is for female and M for male with the accompanying number being the identifier allocated to student or resident participants to maintain confidentiality. The summary of lessons learnt from the pedagogy of partnership that enabled the offering of the EFAR programme in Ocean View, as a student led intervention, is presented as three themes. These are reported as representing one way to respond to the need for authenticity in the learning environment.

### From research to a student led intervention

The words of a community resident: ‘We stand and watch people die because we do not know what to do’ became a key driver to implement the EFAR intervention, as a curriculum activity. There was therefore an organic transition from the CBR where the need (problem) emerged, to the CBSL project.

The data show that students appreciated that they:

‘… gained so much knowledge about being a first responder’ (Participant 8, student, male)

As well as having the opportunity to:

‘… get to know people in this community’ (Participant 6, student, female)

So that they:

‘… now understand much better how things work here and what it is like to live in Ocean View’ (Participant 2, student, female).

This indicates a deeper awareness of the social circumstances of people within the community, while imparting knowledge to the community members. Furthermore, the established SL structure in the EMS department enabled the EFAR programme to be accommodated as a new project within the CBSL options of the EMC curriculum. The students who voluntarily selected this project entered a learning space that they orchestrated to facilitate what can be viewed as reciprocal learning, as demonstrated by the student and resident who said it this way:

‘I am only in second year but being here has shown me that I already know a lot – but of course I still have so much to learn. I think it is nice that I can pass on what I know to people here so they can also help to save lives. I hope they know how much I am also learning from them.’ (Participant 1, student, female)

‘How amazing that I am learning first aid. I thought the students would not be able to teach us, but they can. There is so much information, but they helped us get it. Also, we can teach them things they did not know – it’s my happiest.’ (Participant 24, resident, female)

This intervention required that the researchers were open to emerging community needs and translated this into a learning opportunity. The research partnership had to expand to accommodate this activity by the inclusion of academics and students from EMS and key people in the collaborating institution with the expertise to offer the EFAR programme. A necessary enabler was that the academics were flexible and considered this to be a good option to add as a SL project. The engagement was found to enhance student learning in a context where students reported their unique experiences such as:

‘I had a different experience from everyone else because I was in the kitchen and could talk to everyone as they came for things’. (Participant 9, student, female)

### Deep authentic learning

The data showed that the students could meet the high-level goals of deep learning and becoming more socially aware. One student reflects on the rich learning with the comment:

‘How does one begin to describe a life changing project, not only for participants but for myself as a planner as well. This project opened my eyes to just how a community, despite all odds can band together and demand change … I learnt that giving back isn’t always about money. I was able to expand my compassion, respect and gratitude. During this project, I learnt that life isn’t all about bettering yourself and making money, it is about using your skills and talents to empower people around you’ (Participant 1, student, female)

Deep learning for the students was enabled because they heard directly from the people they will serve as practitioners. An example of the deep sharing of participants is that the community of Ocean View arose out of:

‘… the dreaded force removals in 1968 and I was moved to the then Slangkop. It was my most traumatic experience, because I had to leave my beloved grandma and aunt behind’ (Participant 1, resident, female).

Students personally experienced that in this community:

‘… Poverty is the order of the day’ (Participant 12, resident, female).

This is a place where people:

‘… fear for their lives’ (Participant 2, resident, female)

They fear it because it is:

‘… like a horror movie, when you leave your home you not sure that you will make it home alive, cause you get shot for no reason whatsoever’ (Participant 3, resident, female).

Although the community frustrations come from a position of feeling forgotten, there was the sense of a strong, lingering motivation to:

‘… do what our hands find to do to make Ocean View a better place to live in … with all its shame, drudgery and broken dreams, it is still a beautiful world’ (Participant 4, resident, female)

But:

‘You can’t build a strong community if you not willing to go the extra mile’ (Participant 4, resident, female).

Many residents willingly passed on their insider knowledge of the social context in a way that showed understanding of the contribution they could make to the education of health professionals of the future. They wanted students to know about the challenges their community is facing and the heart within the people of Ocean View:

‘[*G*]ood days and then bad days … Since 2014 we’ve lost 124 people due to gang violence including children as young as six months old … But when Ocean View is in mourning, we stands together.’ (Participant 2, resident, female)

The depth of learning is shown from what a student shared from her experience on the EFAR project:

‘Our first contact session with the community went greater than expected as we automatically connected with the community participants with a roaring and vibrant group of participants. Our following contact session thereafter, was also a great success with more interest in the project that lead to an additional date for a fourth contact session … In conclusion, after all the countless obstacles the project has been a success and a stressful delight … by helping the Ocean View community and being part of the change.’ (Participant 2, student, female)

It is insights such as this experience being a ‘stressful delight’ that show how worthwhile this learning intervention was for the students.

### Learning as a shared experience

We started to recognise the relevance of transferring the ideology of a focus on partnership and shared experience to the curriculum for the CBSL project. Hence, we included undergraduate EMC students and lecturers to partner with the researchers, community members, and the NPO for delivery of the EFAR programme. This mix of partners, with obviously differing pasts, life experiences, and social circumstances worked together in a collaborative entanglement, where all parties learnt together in what emerged as a theme of shared experience enhanced by the asymmetry, as is in evidence from the comments of two participants:

‘I am so ecstatic to be in this project. I am in first year but wanted to experience service learning before I have to do something myself next year. First I wanted to help another group … but this project needed helpers so I volunteered. There are also third years here. It is great being with senior students and learning from them and I am also learning about the people here because they are so OK to share and talk,’ (Participant 5, student, male)

‘There have been other courses – not first aid but other things – this is the best because we are helping the students and they are helping us. This makes me feel good as we are sharing together and getting skills that will be useful’ (Participant 62, resident, male)

And then from a resident who returned to a subsequent training session just to tell us, the potential value of the shared learning experience was reinforced:

‘When I got to know about first aid it gave my life meaning and I have saved a man shot in my street when I was the first to arrive there and keeped [kept] him alive until the ambulance came’ (Participant 5, resident, female).

The EFAR programme was initiated because of the frustrations expressed by community members during a research project and then the students were supported to take the lead in the design and delivery of the activity within this community. The close contact with residents was shown to enable a space where the students became less focused on self:

‘It’s funny that I don’t even think of my own worries when I am here.’ (Participant 7, student, female)

They could think about issues from the perspective of the community:

‘… because now I am thinking about this community and how life is for them.’ (Participant 9, student, female)

They used the information gained to determine if their action was socially just:

‘… and I want to do something to help them in the best way I can’ (Participant 7, student, female).

In this engagement, aimed at a mutually beneficial outcome, the students indicated that they; ‘… enjoyed being here and sharing in a deep and meaningful way’ (Participant 1, student, female) and with this they were able to grow in the journey towards being socially conscious citizens.

The students were able to reach deeper awareness of the society they serve as EMC practitioners, and they now have a more nuanced understanding of how life in one community will impact their practice. In this cycle of participatory research and an intervention involving students, learning was influenced in ways that were not anticipated at the outset and was enhanced through this dynamic and deeply meaningful shared experience.

The researchers, lecturers and students were warmly invited into this community and close bonds were formed that was expressed by one student in this way:

‘Their gratitude towards us is a speechless act because they do not realise what a great and major impact they made on my life and that the amount of gratitude that I have for them are even more then what could ever be comprehended.’ (Participant 2, student, female)

This community could offer students the opportunity for learning through a model of a pedagogy of partnership where existing partnerships were extended for a curriculum intervention of mutual benefit and the respectful understanding of difference.

## Discussion

In the shift towards a relevant curriculum that takes account of cultural practices and the needs of communities, the theoretical lens of AR helped to gain an understanding of the engagement between students, offering and contributing to the EFAR programme, and the recipient community members. In this student led training, there was the opportunity for mutually beneficial engagement (Young 1997). Through reflection and using the lens of AR, the differences and inhomogeneities were recognised as part of this mutually beneficial, reciprocal engagement (Silbert 2019). Some students identified with the community members through similarities such as sharing Afrikaans as their first language or having grown up in another community in Cape Town with some similarities. Still there was a chasm of differences and the need for an overt decision to demonstrate respectful characteristics and strive towards a relationship of equals (Silbert 2019; Trimble & Fisher 2006; Young 2020), which demanded awareness, sensitivity and commitment from the students.

According to Maiter et al. (2008), there are two categories that limit reciprocity. The first are structural limitations with one example being that researchers have no control over the broader societal resources and another that community partners are a limited representation of the diversity in their community. The second category limiting reciprocity are organisational limitations such as the case that university incentives can preclude researchers engaging in community advocacy work and interference in the establishment of reciprocal relationships because of the high turnover of persons in community structures. These reciprocal limitations were in part mitigated against in this project and furthermore the impact of the investment of resources can be loosely measured by meeting the purpose of the training with the several reports of help being given to trauma victims while awaiting the ambulance. Also, there are at least three women who were able to access home care training because of having completed the EFAR programme.

The 72 participants were partially representative of the diversity of the community but importantly they enabled the students to have an authentic engagement in this community. With regard to organisational limitations, although still transitioning to equal recognition of CBR, the university system encourages community involvement and counts SL as important. Then of note is that the relationships built in this community through the extended research period has been consistent and there have not been marked changes.

The explorations of the ways in which the pedagogies and practices in HE can transform HPE was assisted by the national acceptance of the need for undergraduate students to be exposed to SL opportunities in diverse communities. What was shown through this study is that there is a potential flow from CBR to student led community-based interventions that enable deep authentic learning as a shared experience. This means that research in a community can be an effective mechanism to build partnerships that facilitate the identification of relevant community projects for the involvement of undergraduate students.

The involvement of EMC students in the intervention to prepare and present the EFAR training, created a space for learning in an environment where the learning opportunity emerged organically and with social justice as a core value. By engaging directly in a community, the students could appreciate difference, remain respectful, make a contribution by offering their professional expertise and learn from the community, as the experts of local knowledges, practices and cultural understandings (Akhurst et al. 2016). The pedagogy and practice of involving community members, students and academics, learning together in a participatory, reciprocal approach, was aligned to the paradigm of the co-creation of living knowledge (Facer & Enright 2016) that influenced the design of the main IKPST project and facilitated the implementation of the EFAR programme. Through this journey it became apparent that the pedagogical shift of second year students taking responsibility for all aspects of a complex CBSL project, the EFAR programme, allowed them to grow in confidence and develop as reflective practitioners (Schön 1983). They were also able to consolidate their learning through the report back to the class of second year students . In this forum they could share their relevant learning as they too learnt from the other students sharing their diverse SL experiences in other communities.

The key lesson learnt through this study was that the relationships, built through CBR, facilitated a partnership whereby the learning environment of undergraduate students could be influenced to allow them to share their knowledge of first aid. In this curriculum activity, the challenges and hopes of the community of Ocean View were experienced directly by the students, and the community could in turn experience the university as actively working for change. The ground up generation of a community need to be exposed as a frustration by residents of not knowing what to do for injured people, was translated into an appropriate intervention selected as one possible option for a SL project. The EFAR programme offered by students in this way, meant that in their second year of study they were already acting as change agents. The students could therefore experience empowering the community participants by providing them with skills to respond in a medical emergency and in the cycle of reciprocal learning, the students were also empowered by the community participants who passed on knowledge about themselves and their community that cannot be taught in the classroom.

This intervention was consistent with the goals of a WIL pedagogy where the academic and workplace competencies of students are broadened through the integration of work placements and other learning activities (Rambe 2018). The types of WIL (Winberg et al. 2011) cover a wide range of possible curriculum activities that can include community projects. In line with the call for more inclusive and relevant learning spaces (Jacobs et al. 2020), the EFAR programme provided an authentic ‘workplace’ opportunity as a CBSL project for EMC students as an extension to the more typical work placements. In this expanded interpretation of WIL, all students did not have the same experience but each was exposed to an environment of difference and asymmetry that afforded them an opportunity for meaningful engagement and reciprocal learning (Young 2020).

What we have termed, a pedagogy of partnership, requires a careful selection of SL activities in established research sites. The students are able to enter environments because of the established partnership and are enabled to gain meaningful experience in the communities in which they will practice and where their patients will reside or originate from. This pedagogical approach can be replicated through ongoing CBR uncovering needs suited to student led interventions. The interpretation of SL is also expanded to be a more inclusive and dynamic design of CBR informing potential activities. In the process, the benefits accrue to all parties in a more equitable manner, aligned to the ethos of asymmetrical reciprocity. This acknowledges the community as the custodian of local, situated knowledge, and as teachers of the students who through this engagement will become more socially aware citizens and practitioners. However, it relies first on the researchers exposing the need, and lifting it into a place where there can be a student led intervention.

This pedagogy responds to the aspirations of the African Union Commission’s (2015) Agenda 2063: The Africa We Want, in that there is respect for human rights, social justice and development, driven by the people, in this case residents in the community of Ocean View who were involved because of the more equitable design that was able to create spaces for learning opportunities that could emerge quite organically.

Furthermore, the vision of this forward-looking agenda is for the youth of this continent to grow the African knowledges and drive transformation; and in this EFAR programme, the shift was to empower students as trainers and partners in their own learning while contributing to social transformation. In this way the dual aims of: CBR and offering students an opportunity to gain experience in a relevant and authentic setting were laced together. There was a shared creation of knowledge as students learnt how to teach and convey the professional expertise they have to participants who held the contextual knowledge on the type of incidents in their environment where first aid would be helpful and potentially lifesaving. All the dimensions of health science, politics, social justice, mutual benefit, and differing lived experiences were intertwined through interactivity with stakeholders (Mobjörk 2010) engaged in this education intervention. This shrank the distance between university and community and mitigated against the trap of taking from this community without giving anything in return (Wagner, Kawulich & Garner 2012).

In a pedagogy of partnership, the relationships, are based on respectful dialogue, challenge inequalities, aim to be reciprocal and at all times strive to be ethical. The asymmetrical relationships led to a deeper understanding of participants’ life experiences and generated knowledge that can be seen as emancipatory and beneficial to all involved. The benefits of the exchange flowed both ways and while providing skills to community members, our students acquired professional and practical skills as well as increased confidence. They gained skills in critique and reflection and heightened social awareness as they drew lessons from this deep connection with the community in an engagement that allowed them to give back in an authentic way (Akhurst et al. 2016). They learned the value of respect and participatory learning and had a real-time experience of problem solving such as when the projector would not work and they transitioned to demonstrating first aid procedures on each other for the group.

This article offers insights from the perspective of a particular CBSL project that emerged through CBR. It was shown that normative practices can be disrupted to open possibilities in a partnership of difference (Young 2020).

Through this opportunity there was the enhancement of student learning, the empowerment of students as holders of professional knowledge and of community members as the holders of contextual knowledge, within a structure of a pedagogy of partnership. The students had access to established community partners and to community experiences that challenged their preconceptions and allowed them to engage in exchanges of knowledge about relevant issues in the social context (Akhurst et al. 2016). Nurturing the fragile spaces of engagement in the unequal context was enabled through a process that is based on the notion of difference as highlighted in asymmetrical reciprocity (Silbert 2019).

## Conclusion

Within the larger study, focused on safety in the community, there was the simultaneous consideration of student engagement through an intervention that emerged as an opportunity to make a positive difference in Ocean View. There was the creation of a meaningful learning opportunity for students, where they learnt respect for local knowledges and gained enhanced social awareness. In this way we suggest that relationships can be established through CBR that can lead to a pedagogy of partnership between the university and the community. In this partnership the university is viewed as caring and actively working for change, while the community offers shared learning spaces. In this and other similar engagements, the students, as agents of change, can share their knowledge and simultaneously grow as socially aware citizens through experiencing the challenges and hopes of the community. There needs to be monitoring of the intervention and the academic staff were essential partners in the pedagogy of partnership in so far as they provided oversight of the planning and implementation of the activity. This relied on academic staff members who were comfortable with student led CBSL activities. They also contributed to the deep learning enabled through students reflecting on their experience and documenting this in reflective journals that facilitated the learning being brought back into the classroom to be shared.

As Higher Education institutions we need to seek ways to engage more with communities. Through this universities can contribute more to social transformation and offer students opportunities to grow as responsible and socially aware citizens. Our key recommendation is that this engagement can be facilitated through CBR projects being designed to include a second objective of identifying community needs suited to social interventions by students.

In conclusion, we emphasise the success of an education model that relies on a pedagogy of partnership. In this transdisciplinary interaction there was a boundary crossing activity that brought the university and community into a closer relationship. The students, residents, researchers and academic staff were then positioned to develop a relationship of equals where there was reciprocal learning.
